# Effects of Operating Temperature on Droplet Casting of Flexible Polymer/Multi-Walled Carbon Nanotube Composite Gas Sensors

**DOI:** 10.3390/s17010004

**Published:** 2016-12-22

**Authors:** Jin-Chern Chiou, Chin-Cheng Wu, Yu-Chieh Huang, Shih-Cheng Chang, Tse-Mei Lin

**Affiliations:** 1 Department of Electrical and Computer Engineering, National Chiao Tung University, 1001 University Road, Hsinchu City 30010, Taiwan; chiou@mail.nctu.edu.tw (J.-C.C.); s0450737.eed04g@g2.nctu.edu.tw (T.-M.L.); 2 Institute of Electrical Control Engineering, National Chiao Tung University, 1001 University Road, Hsinchu City 30010, Taiwan; yuchieh.ece99g@g2.nctu.edu.tw; 3 Institute of Biomedical Engineering, National Chiao Tung University, 1001 University Road, Hsinchu City 30010, Taiwan; scchang1222.iie04g@g2.nctu.edu.tw

**Keywords:** polymer/multi-walled carbon nanotube composites, droplet casting, operating temperature, impedance spectrum

## Abstract

This study examined the performance of a flexible polymer/multi-walled carbon nanotube (MWCNT) composite sensor array as a function of operating temperature. The response magnitudes of a cost-effective flexible gas sensor array equipped with a heater were measured with respect to five different operating temperatures (room temperature, 40 °C, 50 °C, 60 °C, and 70 °C) via impedance spectrum measurement and sensing response experiments. The selected polymers that were droplet cast to coat a MWCNT conductive layer to form two-layer polymer/MWCNT composite sensing films included ethyl cellulose (EC), polyethylene oxide (PEO), and polyvinylpyrrolidone (PVP). Electrical characterization of impedance, sensing response magnitude, and scanning electron microscope (SEM) morphology of each type of polymer/MWCNT composite film was performed at different operating temperatures. With respect to ethanol, the response magnitude of the sensor decreased with increasing operating temperatures. The results indicated that the higher operating temperature could reduce the response and influence the sensitivity of the polymer/MWCNT gas sensor array. The morphology of polymer/MWCNT composite films revealed that there were changes in the porous film after volatile organic compound (VOC) testing.

## 1. Introduction

Polymer-based sensors are resistive-type gas sensors that are widely used by extant research for gas and vapor sensing owing to their diverse responses to different gases. Polymer composite chemiresistor gas sensor arrays comprise different polymers and carbon particles that interact with an adsorptive analyte and cause electrical property changes. Several previous studies examined the high response and sensitivity of polymer-based sensors for detection of volatile organic compounds [1,2,3]. Carbon nanotubes (CNTs) have stimulated great interest due to their distinctive electrical, physical, and chemical properties that enable the development of sensitive devices in the field of gas sensing [4,5]. Polymer/MWCNT composites have attracted considerable attention due to fast response and high sensitivity towards environmental gases at room temperature. Recent studies demonstrate feasibility of polymer/MWCNT composites for detection of toxic chemical agents, inorganic vapors, and volatile organic compounds [6,7,8,9,10,11,12,13,14].

Operating temperature of the sensing layer is a key factor that affects response time, sensitivity, and the baseline for both metal oxide semiconductor (MOS) gas sensors and polymer-based gas sensors with different absorbed gases. Specifically, an MOS gas sensor equipped with an oxide-based layer that operates at high operating temperatures causes a change in charge mobility during chemisorptions of oxygen [15]. The operating temperature of SnO_2_-based gas sensors that ranges from 25 °C to 500 °C detects various types of low concentration gases [16,17]. Polymer-based gas sensors are characterized by swelling due to the absorption of a target gas into the polymer layer, and subsequently the variation of an electric signal results in a charge transfer on the surfaces of the CNTs [8,18]. The operating temperature of polymer-based gas sensors corresponds to low temperatures below 80 °C to guarantee a stable response to mitigate the influence of ambient temperature [19,20,21]. 

Polymer-carbon black composite gas sensor arrays that are operated at low temperatures were developed and applied in electronic nose systems [19,20]. Extant research has examined resistance changes with respect to varying ambient temperatures in polymer-carbon black films in detail [21,22]. The results indicated that the ambient temperature could influence the resistance and baseline at different molecular weights and different carbon loadings [23]. The results of previous studies indicated that different polymer-carbon black composite gas sensors manipulated at several low operating temperatures could exhibit a decrease in their response to the target gases as the operating temperature is increased [24,25,26]. Many reports on MWCNT/polymer based gas sensors demonstrated high sensitivity but slow recovery at room temperature to achieve complete desorption of adsorbed gas molecules from the surface of MWCNTs. Thermal treatment is one of the more efficient methods to tackle the poor recovery [27,28,29]. Nevertheless, our preliminary study had shown that the sensing response of a flexible polymer/MWCNT composite gas sensor was decreased with increasing operating temperature [30]. Extant studies have not examined the effect of temperature on the polymer-carbon black composite sensors, with respect to mechanisms of tunneling, hopping, and thermal expansion. Additionally, it is not fully understood how a variation in temperature affects the electrical properties of polymer/carbon nanotube composite gas sensors and causes different chemical potentials of polymer phase and gas phase.

In this study, a two-layer polymer/MWCNT composite sensing film was fabricated by a droplet casting method, and a flexible printed circuit (FPC) technology was used to fabricate sensing electrodes with embedded heater gas sensor arrays. The operating temperature dependence of electrical characterization and sensor response was investigated. The selected polymers used in a polymer/MWCNT composite sensing film included ethylcellulose (EC), polyethylene oxide (PEO), and polyvinylpyrrolidone (PVP). The effect of different operating temperatures on the electric properties and sensing responses of the polymer/MWCNT composite gas sensor array was tested in the device developed to detect methanol. Furthermore, scanning electron microscopy (SEM) was used to compare differences in morphologies between the sensors before and after the test.

## 2. Materials

The polymer/MWCNT composite sensing film consisted of two membranes, the top layer and the bottom layer wherein the polymer film and the MWCNT film were deposited, respectively. Both membranes were fabricated via a droplet casting method to form the two-layer structure for gas sensing. Polymers selected for deposition on the MWCNT film included ethylcellulose (200679, Sigma-Aldrich, Saint Louis, MO, USA), polyethylene oxide (43678, Alfa Aesar, Haverhill, MA, USA), and polyvinylpyrrolidone (PVP 10, Sigma-Aldrich, Saint Louis, MO, USA). The selection was based on linear solvation energy relationship (LSER) theory and physical absorption bonding [31,32]. Typically, each of the selected polymers (0.2 g) was dissolved in 20 mL tetrahydrofuran (THF) and was then prepared by sonication for 6 h in an ultrasonic bath at room temperature. The MWCNT used for the composite films were few-walled carbon nanotubes (FWNTs) provided by the XinNano Materials, Inc. (Taoyuan, Taiwan). The approximate dimensions of the MWCNT with 2–5 layers of sidewalls were an average diameter of 4 nm, 10–12 μm average length, and >86% average purity.

**Figure 1:**
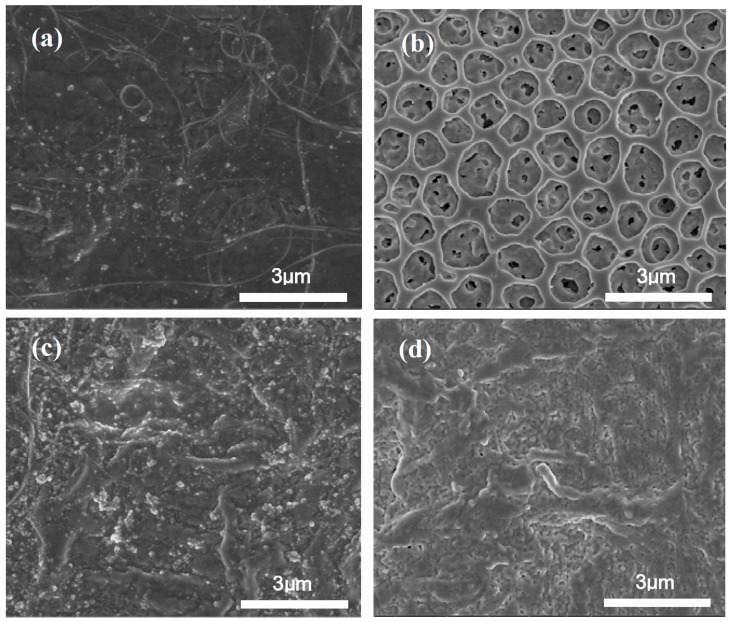
SEM morphology of polymer/MWCNT composite films before the test (**a**) MWCNT film; (**b**) EC/MWCNT film; (**c**) PEO/MWCNT film; and (**d**) PVP/MWCNT film.

The fabrication processes of droplet casting a two-layer sensing film are as follows: first, 1 wt % (2 μL) of MWCNT was deposited on a conductive electrode by a micro jet. The device was then placed in an oven at 70 °C to evaporate THF and furnish the MWCNT film. The selected polymers were then deposited by adding a droplet of 1 wt % (2 μL) solution (1 mg/mL THF) on the MWCNT layer to form the film. Finally, the device was dried for 24 h at 60 °C, and the solvent was completely evaporated prior to use. The sensor resistance after each casting step was monitored to limit the value within a range of 10 kΩ–200 kΩ to guarantee the reproducibility. The morphology of all polymer/MWCNT composite films was confirmed by the SEM image as shown in Figure 1a–d. The morphology of a polymer sensing film was examined using an SEM (NOVA NANO SEM 450, FEI Co., Hillsboro, OR, USA) with 10 kV acceleration voltage. The pore sizes of EC/MWCNT film remained in a range of 0.7–1.1 µm. The pores with the largest diameter were in the range from 1.2 µm to 1.4 µm.

## 3. Design and Fabrication

**Figure 2:**
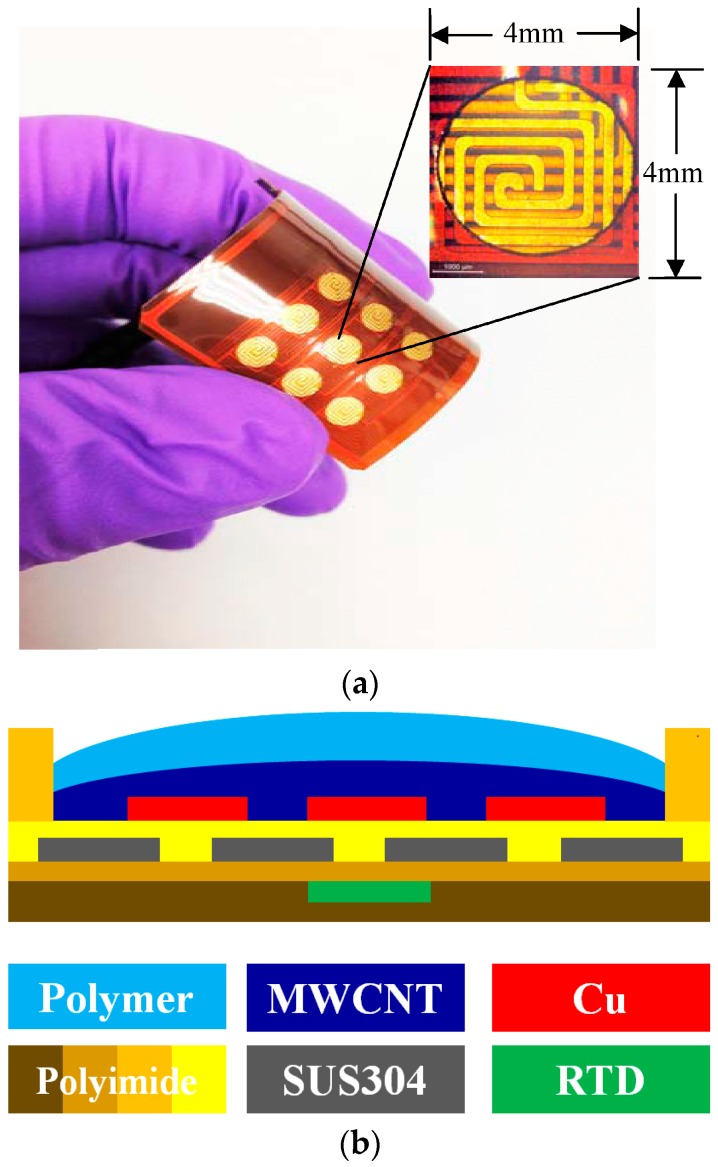
(**a**) configuration of the flexible gas sensor array. The inset shows the sensing electrode (**top** electrode) and the heater (**bottom** electrode); and (**b**) the cross-sectional schematic structure of the single gas sensor.

A cost-effective gas sensor array was fabricated by flexible printed circuit industry technologies. The flexible gas sensor array was comprised of three different types of polymer/MWCNT composite sensing films arranged in a 3 × 3 matrix pattern. Each type of the selected polymer was arranged in one of the rows in the matrix. The fabricated flexible gas sensor array exhibited excellent flexibility, as shown in Figure 2a. The insets indicate the sensing electrode of a single sensor element and the heater.

The sensing electrode was composed of copper with 35 μm thickness, 220 μm line width, and 220 μm line spacing. The through hole-machined well with 130 μm thickness was positioned and then adhered to the upper side of the sensing electrode to guarantee a filled polymer composite film placed in a specific area [33]. The configuration of the fabrication and cross-section view of the flexible gas sensor array is shown in Figure 2b.

The heater had a 50 μm thickness and a geometry corresponding to 20 mm × 20 mm. In contrast, the width and spacing of the single heater line was 220 μm and 280 μm, respectively. The heater was made of stainless steel (SUS304) to provide a thermostat operating temperature. These temperatures included room temperature, 40 °C, 50 °C, 60 °C, and 70 °C. The platinum resistance temperature detector (RTD) was embedded in a polyimide substrate to enable feedback control at the operating temperature. To prevent heat loss from the substrate, 130 μm polyimide films were adhered to the bottom side of the sensor substrate. Both the heater as well as the sensing electrode were designed in double-spiral shapes in a square area [30].

**Figure 3:**
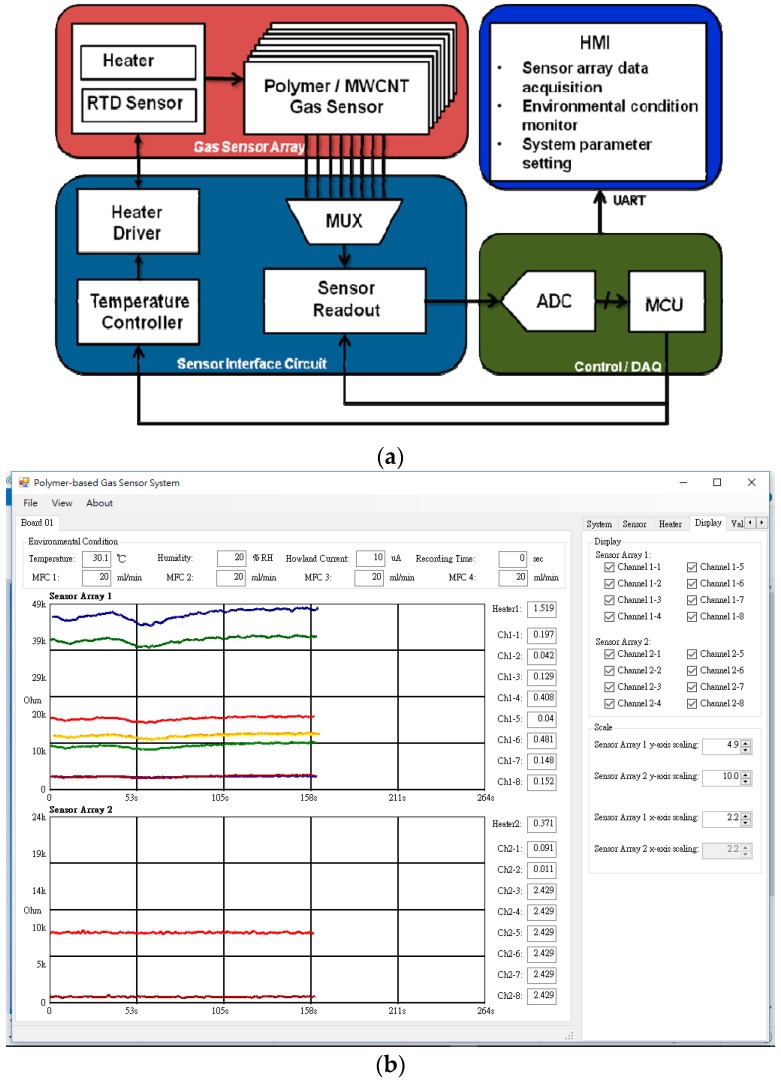
(**a**) system block of the gas sensor array control system; and (**b**) human–machine interface software.

The architecture of the gas sensor array control system was comprised of a flexible gas sensor array sensor, an interface circuit, a micro control unit, and a human–machine interface. The system was designed to drive the sensor array and the heater, and to control the operating temperature and collect response data from each sensor. Figure 3 shows a block diagram of the proposed gas sensor array control system.

When a flexible gas sensor array was operated at a specific temperature for target gas detection, the varied resistance of each sensor was obtained through a multiplexer (MUX). The resistance was then converted to voltage signals by a sensor interface circuit (SIC). The multichannel signals were recorded through a micro control unit (MCU, C8051F120, Silicon Laboratories, Inc., Austin, TX, USA) and then synchronized display was obtained on the human–machine interface (HMI, see Figure 3b).

**Figure 4:**
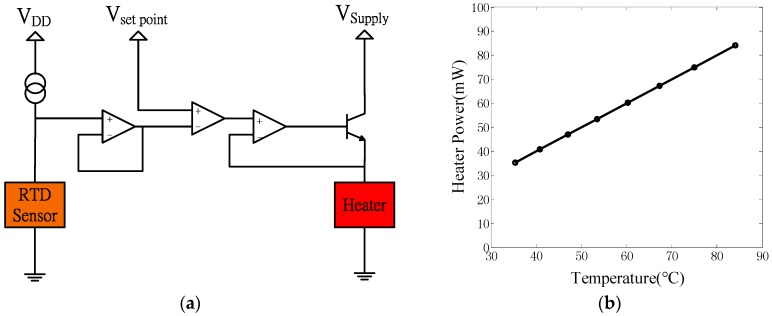
(**a**) temperature control circuit of the heater; and (**b**) power consumption vs. operating temperature of the microheater for the flexible gas sensor array.

The driver and feedback control circuit of the heater are shown in Figure 4a. The operational amplifier was connected to a voltage source and operated a bipolar junction transistor that functioned as a switch for the current to the heater [34,35]. The 1 kΩ platinum RTD sensor that measured the change in the operating temperature of the heater was driven by a constant current source. The signal of the RTD sensor was obtained by a voltage follower and was then connected to one of the inputs in the differential amplifier in the compensator circuit. This output signal was compared to the reference temperature set-point voltage for driving the heater. This feedback control system for the heater was used to obtain the steady-state electrical power consumption curves of the heater under 500 mL/min airflow conditions given the existence of the composite-sensing layer, as shown in Figure 4b. The electrical power consumption of the heater was a function of the operating temperature range (35.36–84.03 °C) in a flexible gas sensor array.

## 4. Experiments and Discussion

### 4.1. Transient Response of the Polymer/MWCNT Composite Film

**Figure 5:**
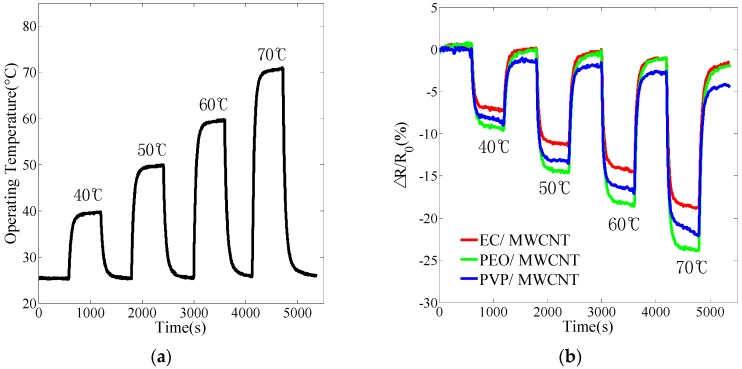
Cyclical heating to operating temperatures of 40 °C, 50 °C, 60 °C, and 70 °C. (**a**) the transient response of the heater to the relative operating temperature; and (**b**) the responses of normalized resistance of the polymer/MWCNT composite sensor.

The transient response experiment included four separate heating stages to operate the microheater by heating the flexible gas sensor array to temperatures of 40 °C, 50 °C, 60 °C, and 70 °C under a 500 mL/min airflow condition. In each stage, the flexible gas sensor array was first maintained at room temperature to obtain the recovery baseline for 10 min. The heater was then used to heat the sensor array to the specific operating temperature for 10 min. Figure 5a shows the transient response of the heater to the relative operating temperature. The profile display indicated that the time taken to heat the array to the operating temperature range (±0.5 °C) using the heater was less than 2 min.

The transient responses of three different polymer/MWCNT composite films are shown in Figure 5b. Evidently, the recovery baselines of the EC/MWCNT and PEO/MWCNT composite films were slightly shifted and PVP/MWCNT was heavily shifted. Three different polymer/MWCNT composite films exhibited a negative temperature coefficient resistance (NTC) inclination as the operating temperature increased. 

With respect to different operating temperatures, the EC/MWCNT and PEO/MWCNT film revealed a better immunity to temperature influence with a variation in resistance The PEO/MWCNT film revealed the widely transient response with respect to the operating temperature that was considerably more stable than other films. The resistance of the PVP/MWCNT film was stable at 40 °C and 50 °C, but unstable at 60 °C and 70 °C. 

### 4.2. Impedance Spectrum Property

**Figure 6:**
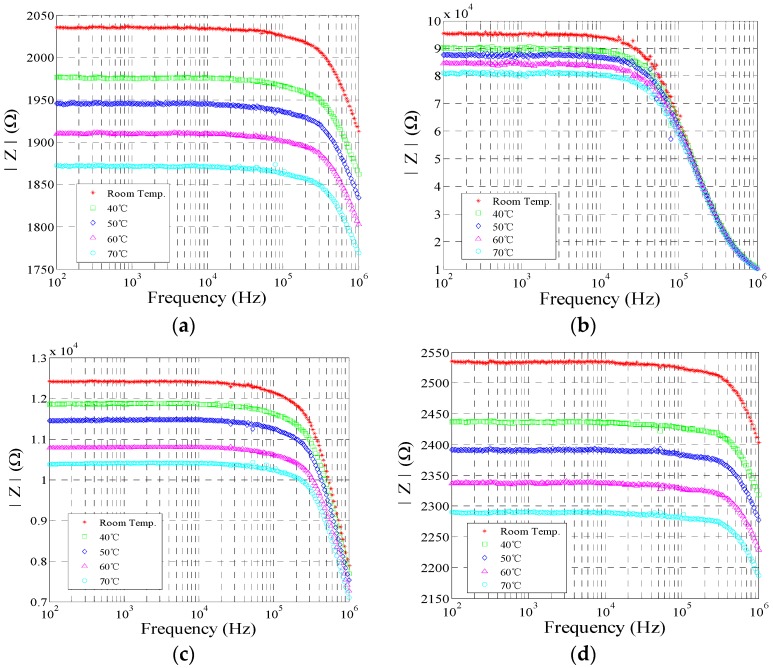
Impedance spectrum of the MWCNT film and polymer/MWCNT composite film at different operating temperatures. (**a**) MWCNT film; (**b**) EC/MWCNT film; (**c**) PEO/MWCNT; and (**d**) PVP/MWCNT.

Impedance measurement was performed using an Agilent 4292A impedance analyzer (Agilent Technologies, Santa Clara, CA, USA)in the frequency range of 100 Hz to 1 MHz using a modulation voltage of 500 mV (peak to peak) [4,36]. The impedance measurements were measured at different operating temperatures under the 500 mL/min airflow conditions. The impedance spectrum of the MWCNT film and three different polymer/MWCNT composite films are shown in Figure 6. The sensor responses were comparable, as shown in the figure.

As the measurements indicate, the resistance behavior of MWCNT film decreased with increases in operating temperatures at frequencies below 100 kHz, corresponding to the operating temperatures (the following operating temperatures: room temperature, 40 °C, 50 °C, 60 °C, and 70 °C). The equivalent circuit model of the polymer/MWCNT composite film was examined. It included two components, namely the resistance and capacitance effects [4]. Significant differences in the behavior of the impedance spectrum were not observed in the other polymer/MWCNT composite films. The impedance spectra of the other polymer/MWCNT composite films revealed a circuit model equivalent to that of the MWCNT film. However, the capacitance effect of each polymer/MWCNT composite film occurred in a different frequency range. As observed in Figure 6, the resistance effects of the EC/MWCNT and PEO/MWCNT composite films were observed below 10 kHz. Additionally, the resistance effect of the PVP/MWCNT composite film was observed below 100 kHz.

### 4.3. The Response of the Sensor Array

**Figure 7:**
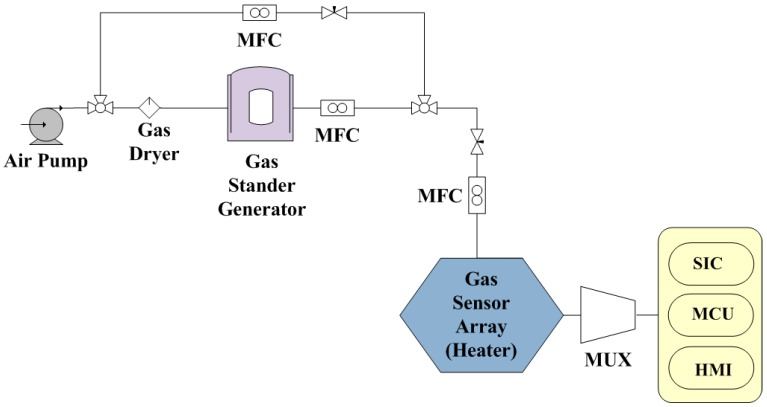
Experimental setup of the gas sensing system.

The polymer composite film absorbed the target gas when it was introduced into the reaction chamber. As the gas was introduced, the film swelled up slightly, and this induced the change in the distance between nanoparticles. The change in the resistance of the film could then be measured by an instrument [8,18]. The experimental setup used in the measurements is shown in Figure 7.

The 1.5% of ethanol gas was controlled by a mass flow controller under a flow rate of 500 mL/min. Dry air (25 °C, 45% relative humidity (RH)) was used as background gas, and the flow rate was set at 500 mL/min. The flexible polymer/MWCNT composite gas sensor array was placed inside a reaction chamber with 60 mL capacity. The gas sensing response measurement consisted of several steps in each gas-testing cycle. First, the heater was heated to the operating temperature, and then dry air was introduced into the reaction chamber for 10 min to obtain a reference resistance baseline. When the temperature of the heater was stable, the ethanol gas was introduced into the reaction chamber for 5 min. The polymer films were adsorbed and swollen due to gas molecules. Following this, dry air was introduced for 10 min to enable desorption from the polymer film. 

**Figure 8:**
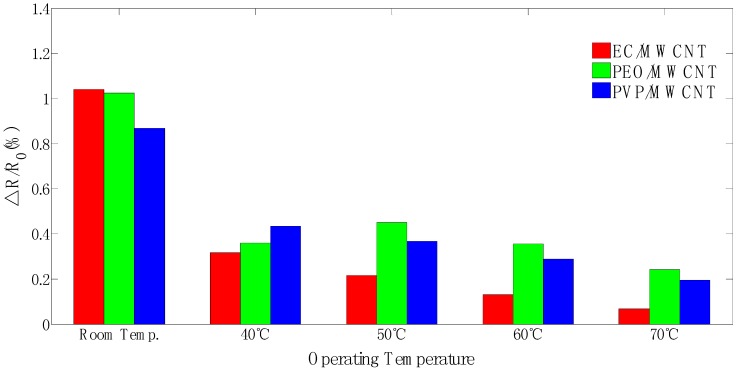
Normalized resistance response of polymer/MWCNT composites films exposed to 1.5% of ethanol gas with respect to different temperatures.

Normalized resistance changes (ΔR/R_0_%) of the polymer/MWCNT composite films were determined using ΔR/R_0_% = [(R_max_ − R_0_)/R_0_] × 100, where R_0_ denotes the mean value of sensor resistance from t = 1~100 s when the sensor was exposed to dry air in equilibrium, and R_max_ denotes the maximum resistance when the sensor was exposed to ethanol. In order to obtain sufficient response information to analysis, the polymer/MWCNT composite sensing film was exposed to 1.5% ethanol with different operating temperatures. Figure 8 shows the response patterns of the normalized data when the polymer/MWCNT composite sensing film was exposed to ethanol with different operating temperatures. The response patterns exhibited that EC/MWCNT and PVP/MWCNT sensors show a decreased response of sensitivity with an increase in operating temperature. Increasing operating temperature could result in increased polymer chain mobility to form percolation networks for sensing response, but simultaneously provide the electrons more energy to overcome the potential barrier and cause more tunneling contribution to decrease the sensing response [37]. The glass transition temperature of PEO is very low and an increase in temperature could result in increased polymer chain mobility at higher operating temperatures [23,37,38]. Hence, the sensitivites of the PEO sensor at 60 °C and 70 °C have better responses than at 50 °C.

All three polymer/MWCNT composite films showed a decrease in sensitivity response with an increase in the operating temperature. The results indicated that the polymer chain mobility increased with an increase in operating temperature with respect to sensing response. A suitable operating temperature could provide a flat baseline for target gas recognition. The baseline shift was severe in the PEO/MWCNT film because this film involves a lower glass transition temperature material that could be sensitive to the operating temperatures.

### 4.4. SEM Morphology of Polymer/MWCNT Composite Films

**Figure 9:**
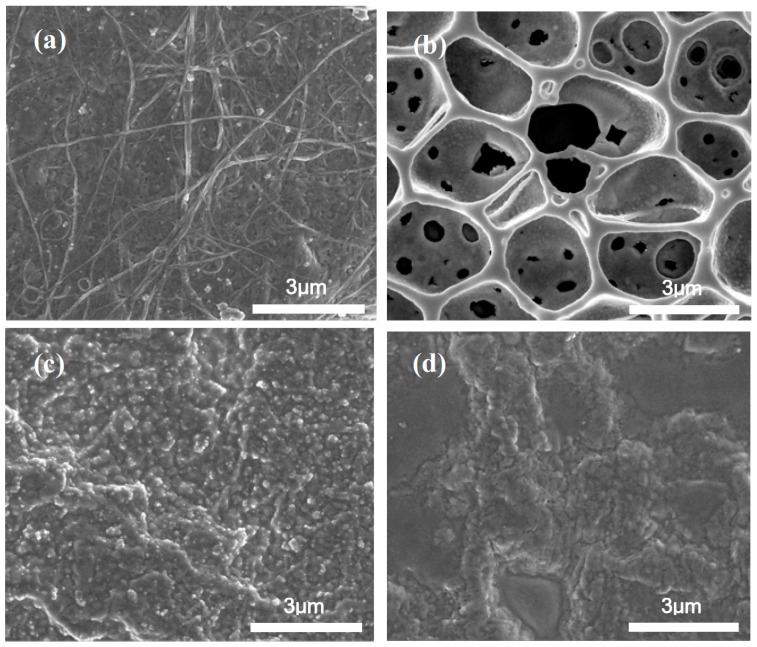
SEM morphology of polymer/MWCNT composite films after the test (**a**) MWCNT film; (**b**) EC/MWCNT film; (**c**) PEO/MWCNT film; and (**d**) PVP/MWCNT film.

The morphology of each polymer/MWCNT composite film after the aforementioned test was investigated via SEM and is shown in Figure 9. The significant differences of the porous EC/MWCNT film indicated that the pores evidently expanded and became larger when compared to the initial pore sizes. The pore size range corresponded to 2.3–2.8 μm with cavity sizes in the range of 0.3–2.1 μm. The surface of the PVP/MWCNT film could shrink after the application of a series of thermal cycles. There were no obvious changes in the other two films after the aforementioned test.

## 5. Conclusions

A flexible polymer/MWCNT gas sensor offers several advantages including cost effectiveness, lower power consumption, reproducibility, lightweight, and flexibility given its potential integration in electronic noses and portable consumer products. However, the ambient environments of these applications involve several variables that influence sensor performance. Temperature is one such variable in which temperature variations pose a critical problem for reducing the sensitivity of the sensor. To date, extant research has not focused on the effect of operating temperature on a polymer-based gas sensor. Current studies examine environments with a constant operating temperature.

The gas absorptions and interaction mechanisms of polymer/MWCNT composite films are dominated by two principles: namely, physisorption and chemisorption. Both of these principles could change with respect to different operating temperatures. The experiment in this study investigated the effect of operating temperature on the responses of a flexible polymer/MWCNT gas sensor. The results indicated that higher operating temperature could mitigate the influence of ambient temperature but reduce the response. Both of these effects could influence the sensitivity of the polymer/MWCNT gas sensor array. The morphology after the aforementioned test showed that the pores of EC/MWCNT expanded, but the surface of PVP/MWCNT film started to shrink. The reusability and the life cycle of each polymer/MWCNT composite film should be considered at a suitable operating temperature to prevent thermal expansion and subsequent destruction of the pores. A future study will examine the effect of other ambient variables and the performance under mechanical strain on the flexible polymer/MWCNT gas sensor array.
